# Transcriptome data on salivary lipocalin family of the Asiatic *Triatoma rubrofasciata*

**DOI:** 10.1016/j.dib.2020.105647

**Published:** 2020-04-30

**Authors:** Daiki Mizushima, Ahmed Tabbabi, Daisuke S. Yamamoto, Le Trung Kien, Hirotomo Kato

**Affiliations:** a Division of Medical Zoology, Department of Infection and Immunity, Jichi Medical University, Japan; b Department of Experimental Chemistry, National Institute of Malariology, Parasitology and Entomology, Vietnam

**Keywords:** Triatoma rubrofasciata, Salivary gland, Lipocalin, RNA-Seq, Transcriptome

## Abstract

The dataset in this report is related to the research article entitled: “Salivary gland transcriptome of the Asiatic *Triatoma rubrofasciata*” [1]. Lipocalin family proteins were identified as the dominant component in *T. rubrofasciata* saliva, and phylogenetic analysis of the salivary lipocalins resulted in the formation of five major clades (clade I-V). For further characterization, each clade of *T. rubrofasciata* lipocalin was subjected to alignment and phylogenetic analyses together with homologous triatomine lipocalins: procalin, a major allergen in *T. protracta* saliva and its homologue Td04 from *T. dimidiata* (clade I), pallidipin and triplatin, inhibitors of collagen-induced platelet aggregation identified from *T. pallidipennis* and *T. infestans*, respectively, and their homologue Pc20 identified from *Panstrongylus chinai* (clade II), Td30 and Td38 from *T. dimidiata* with unknown functions (clade III), triatin-like salivary lipocalins, Pc58 and Pc226 identified from *P. chinai* and Td18 from *T. dimidiata* (clade IV), and triafestin, an inhibitor of the activation of the kallikrein–kinin system, identified from *T. infestans* saliva and its homologues, Td25 and Td40 from *T. dimidiata* and Pc64 from *P. chinai* (clade V).

**Specifications Table**

**Table:** 

**Subject**	Insect Science
**Specific subject area**	Salivary lipocalins of a hematophagous insect
**Type of data**	Table, figure
**How data were acquired**	RNA-seq was performed using HiSeq 2500 (Illumina) with 100-bp paired-end reads. The trinity sequences were aligned with CLUSTAL W software and examined using Molecular Evolutionary Genetics Analysis (MEGA) ver. 6. Phylogenetic trees were constructed by the maximum likelihood (ML) method with the distance algorithms available in the MEGA package
**Data format**	Raw
**Parameters for data collection**	*Triatoma rubrofasciata* specimens were captured in Hanoi, Vietnam. Salivary glands were dissected from adult insects after 2 weeks of feeding, and total RNA was extracted from 20 sets of the salivary glands using NucleoSpin RNA Plus (Takara Bio, Shiga, Japan).
**Description of data collection**	The quality of paired-end reads obtained by HiSeq sequencing was checked by FastQC. All reads were trimmed using Trimmomatic to obtain high-quality sequences, and *de novo* assembly of trimmed reads was performed using Trinity. Read counts and FPKM (fragments per kilobase of exon per million mapped fragments) were calculated using RSEM (RNA-Seq by Expectation-Maximization). CDS were extracted using nucleotide sequence databases of the National Center of Biological Information (NCBI), and their deduced amino acid sequences were analyzed using the non-redundant (NR) protein sequence database of the NCBI, eggNOG orthology prediction database of the European Molecular Biology Laboratory (EMBL), gene ontology database (GO), SWISS-PROT Protein Knowledgebase, and Pfam protein domain database. Putative secreted proteins were identified using the SignalP server.
**Data source location**	Jichi Medical University Shimotsuke City, Tochigi, Japan 36°23′N and 139°51′E
**Data accessibility**	The raw sequencing data has been deposited in DDBJ Sequencing Read Archive under the accession number DRR205094 (https://ddbj.nig.ac.jp/DRASearch/run?acc=DRR205094). The sequence data of trinity transcripts are available in the DDBJ/EMBL/GenBank databases (http://getentry.ddbj.nig.ac.jp/) under the accession numbers ICPO01000359-ICPO01000367, ICPO01000369-ICPO01000421, ICPO01000643, and ICPO01000676. Accession number and direct link of each molecule are shown in Supplementary Table 1.
**Related research article**	D. Mizushima, A. Tabbabi, D.S. Yamamoto, L.T. Kien, H. Kato, Salivary gland transcriptome of the Asiatic *Triatoma rubrofasciata*. Acta Trop. In press.

**Value of the Data**

• The data represents the first report of salivary lipocalins from an Asiatic triatomine bug.
• The results will provide further information on the salivary biochemical and pharmacological complexity of triatomine bugs and the evolution of salivary components in blood-sucking arthropods.
• cDNAs and recombinant proteins prepared from these transcripts will promote the discovery of novel pharmacologically active compounds, as well as the development of biomarkers following exposure to *Triatoma rubrofasciata*.

## 1 Data Description

**Fig. 1:**
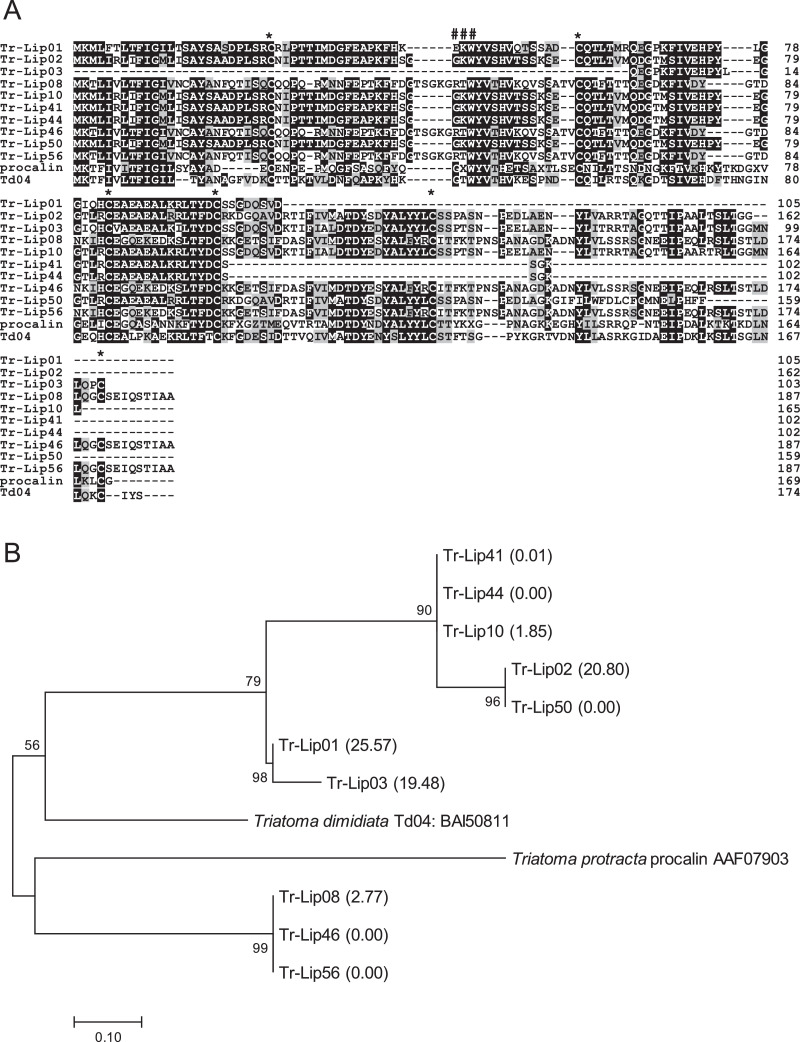
(A) Sequence alignment of procalin-like molecules from *Triatoma rubrofasciata* (Tr-Lip01-03, Tr-Lip08, Tr-Lip10, Tr-Lip41, Tr-Lip44, Tr-Lip46, Tr-Lip50, and Tr-Lip56) together with procalin (accession number: AAF07903), a major allergen in *T. protracta* saliva, and its homologue Td04 (BAI50811) from *T. dimidiata*. Black-shaded amino acids represent identical amino acids, and gray-shaded amino acids represent conserved amino acids. Dashes indicate gaps introduced for maximal alignment. Asterisks at the top of the amino acids denote conserved cysteine residues, and the GXW motif is indicated by ###. (B) Phylogenetic analysis of procalin-like proteins from *T. rubrofasciata* together with procalin and Td04. The numbers in parentheses indicate %FPKM. The scale bar represents 0.1% divergence. Bootstrap values are shown above or below branches.

**Fig. 2:**
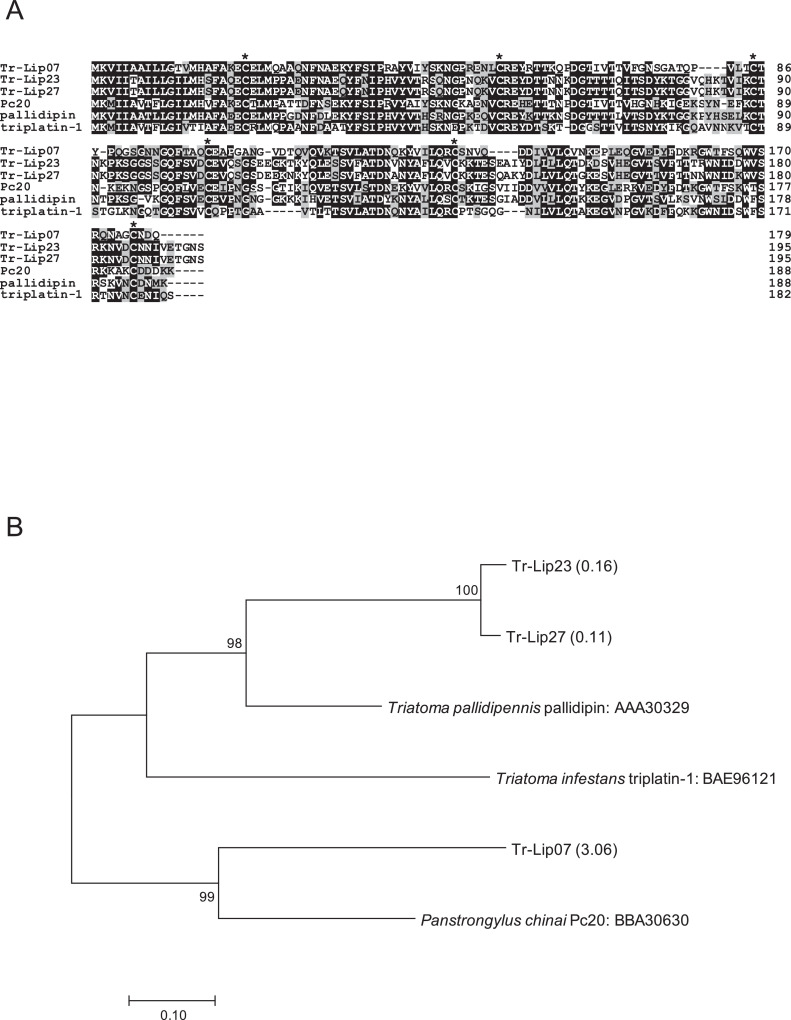
(A) Sequence alignment of pallidipin-like molecules from *Triatoma rubrofasciata* (Tr-Lip07, Tr-Lip23, and Tr-Lip27) together with pallidipin (accession number: AAA30329) and triplatin (BAE96121), inhibitors of collagen-induced platelet aggregation identified from *T. pallidipennis* and *T. infestans*, respectively, and their homologue Pc20 (BBA30630) identified from *Panstrongylus chinai*. Black-shaded amino acids represent identical amino acids, and gray-shaded amino acids represent conserved amino acids. Dashes indicate gaps introduced for maximal alignment. Asterisks at the top of the amino acids denote conserved cysteine residues. (B) Phylogenetic analysis of pallidipin-like molecules from *T. rubrofasciata* together with pallidipin, triplatin, and Pc20. The numbers in parentheses indicate %FPKM. The scale bar represents 0.1% divergence. Bootstrap values are shown above or below branches.

**Fig. 3:**
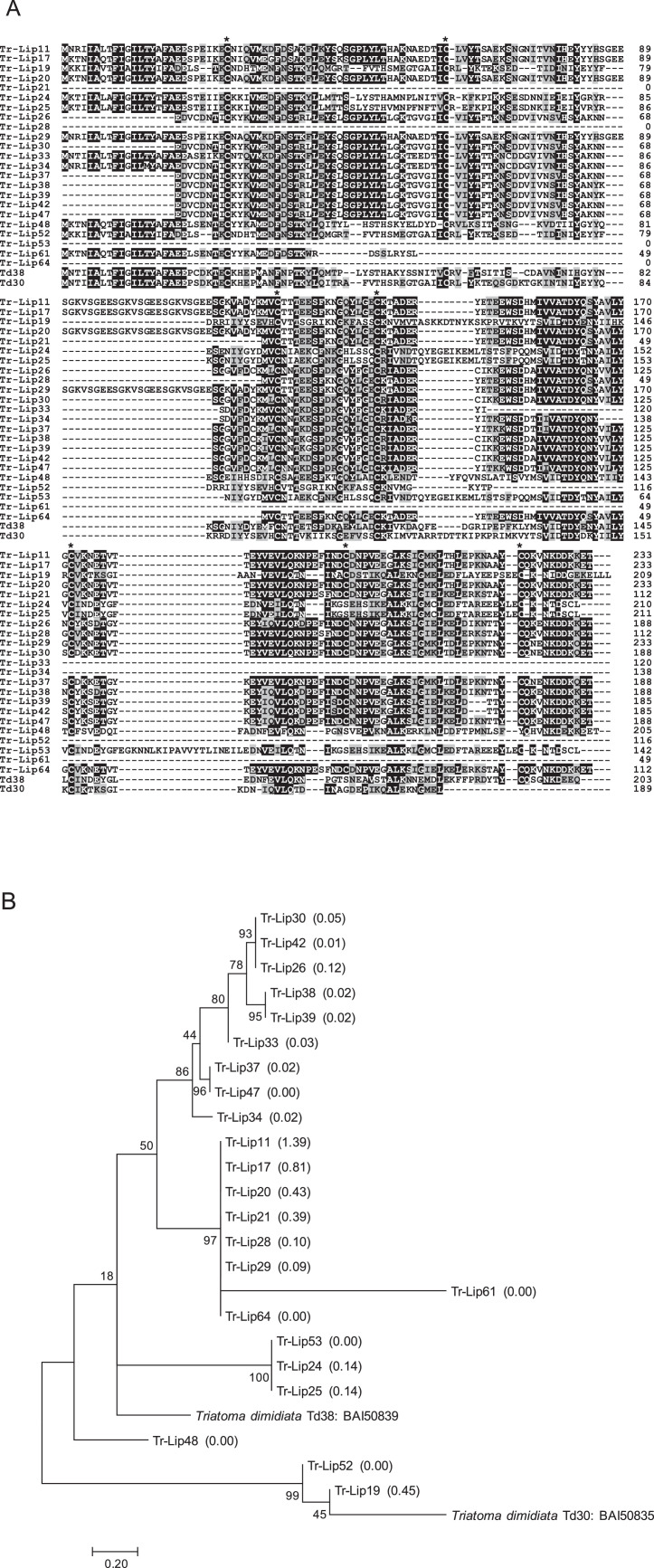
(A) Sequence alignment of Td38-like molecules from *T. rubrofasciata* (Tr-Lip11, Tr-Lip17, Tr-Lip19, Tr-Lip20, Tr-Lip21, Tr-Lip24, Tr-Lip25, Tr-Lip26, Tr-Lip28, Tr-Lip29, Tr-Lip30, Tr-Lip33, Tr-Lip34, Tr-Lip37-39, Tr-Lip42, Tr-Lip47, Tr-Lip48, Tr-Lip52, Tr-Lip53, Tr-Lip61, and Tr-Lip64) together with Td30 (accession number: BAI50835) and Td38 (BAI50839) identified from *T. dimidiata* with unknown functions. Black-shaded amino acids represent identical amino acids, and gray-shaded amino acids represent conserved amino acids. Dashes indicate gaps introduced for maximal alignment. Asterisks at the top of the amino acids denote conserved cysteine residues. (B) Phylogenetic analysis of Td38-like molecules from *T. rubrofasciata* together with Td30 and Td38. The numbers in parentheses indicate %FPKM. The scale bar represents 0.2% divergence. Bootstrap values are shown above or below branches.

**Fig. 4:**
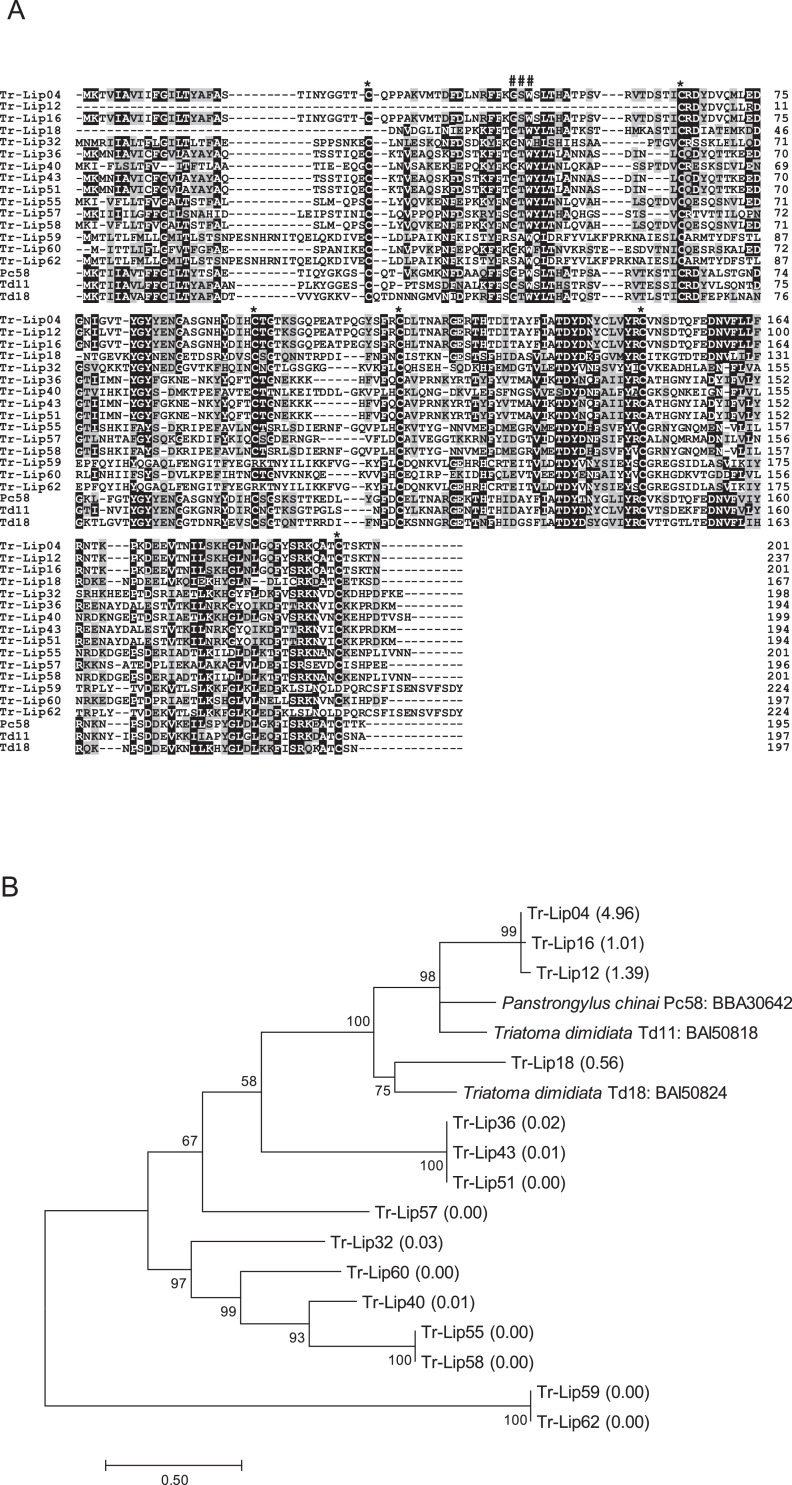
(A) Sequence alignment of triatin-like molecules from *Triatoma rubrofasciata* (Tr-Lip04, Tr-Lip12, Tr-Lip16, Tr-Lip18, Tr-Lip32, Tr-Lip36, Tr-Lip40, Tr-Lip43, Tr-Lip51, Tr-Lip55, Tr-Lip57-60, and Tr-Lip62) together with Td11 (accession number: BAI50818) and Td18 (BAI50824) identified from *T. dimidiata,* and Pc58 (BBA30642) from *Panstrongylus chinai*. Black-shaded amino acids represent identical amino acids, and gray-shaded amino acids represent conserved amino acids. Dashes indicate gaps introduced for maximal alignment. Asterisks at the top of the amino acids denote conserved cysteine residues, and the GXW motif is indicated by ###. (B) Phylogenetic analysis of triatin-like proteins from *T. rubrofasciata* together with Td11, Td18, and Pc58. The numbers in parentheses indicate %FPKM. The scale bar represents 0.5% divergence. Bootstrap values are shown above or below branches.

**Fig. 5:**
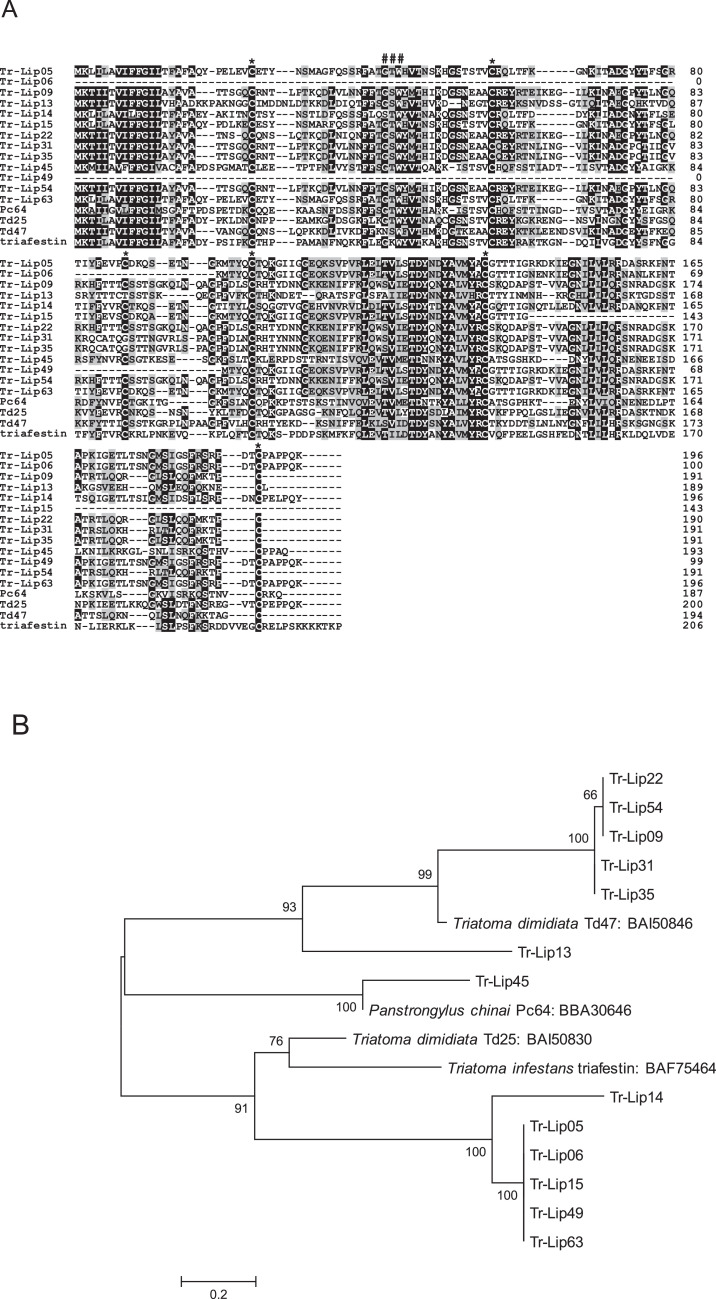
(A) Sequence alignment of triafestin-like molecules from *Triatoma rubrofasciata* (Tr-Lip05, Tr-Lip06, Tr-Lip09, Tr-Lip13-15, Tr-Lip22, Tr-Lip31, Tr-Lip35, Tr-Lip45, Tr-Lip49, Tr-Lip54, and Tr-Lip63) together with triafestin (accession number: BAF75464) identified from *Triatoma infestans*, and its homologues, Td25 (BAI50830) and Td47 (BAI50846) from *T. dimidiata,* and Pc64 (BBA30646) from *Panstrongylus chinai*. Black-shaded amino acids represent identical amino acids, and gray-shaded amino acids represent conserved amino acids. Dashes indicate gaps introduced for maximal alignment. Asterisks at the top of the amino acids denote conserved cysteine residues, and the GXW motif is indicated by ###. (B) Phylogenetic analysis of triafestin-like molecules from *T. rubrofasciata* together with triafestin, Td25, Td47, and Pc64. The numbers in parentheses indicate %FPKM. The scale bar represents 0.2% divergence. Bootstrap values are shown above or below branches.

The salivary gland transcriptome of *Triatoma rubrofasciata* revealed 64 coding sequence (CDS) coding for lipocalin family proteins, which accounted for 89.27% FPKM of the secreted class and 64.82% FPKM of total molecules in the salivary glands [1]. Table 1 shows the grouping of transcripts coding for lipocalin family proteins in *T. rubrofasciata* salivary glands obtained by phylogenetic analysis [1]. Fig. 1, Fig. 2, Fig. 3, Fig. 4, Fig. 5 represent alignment and phylogenetic analyses of each clade of *T. rubrofasciata* salivary lipocalins together with homologous proteins: procalin, a major allergen in *T. protracta* saliva and its homologue Td04 from *T. dimidiata* (clade I), pallidipin and triplatin, inhibitors of collagen-induced platelet aggregation identified from *T. pallidipennis* and *T. infestans*, respectively, and their homologue Pc20 identified from *Panstrongylus chinai* (clade II), Td30 and Td38 from *T. dimidiata* with unknown functions (clade III), triatin-like salivary lipocalins, Pc58 and Pc226 identified from *P. chinai* and Td18 from *T. dimidiata* (clade IV), and triafestin, an inhibitor of the activation of the kallikrein–kinin system, identified from *T. infestans* saliva and its homologues, Td25 and Td40 from *T. dimidiata,* and Pc64 from *P. chinai* (clade V), showing their structural similarity and diversity.

**Table 1:** Trinity transcripts coding for lipocalin family proteins in *Triatoma rubrofasciata.*

Clade	Similar to	No. of CDS	FPKM	%FPKM
Clade I: Procalin-like				
	Td04 (*Triatoma dimidiata*): BAI50811	1	211,532.33	25.57
	Td08 (*Triatoma dimidiata*) : BAI50815	4	176,618.39	21.35
	Td06 (*Triatoma dimidiata*): BAI50813	2	172,131.87	20.80
	salivary lipocalin 2 (*Triatoma brasiliensis*): ABH09420	3	22,910.46	2.77
Clade II: Pallidipin-like				
	Pc20 (*Panstrongylus chinai*): BBA30630	1	25,283.63	3.06
	pallidipin 2 (*Meccus pallidipennis*): AAA30329	2	2,220.47	0.27
Clade III: Td38-like				
	Td38 (*Triatoma dimidiata*): BAI50839	13	22,909.62	2.77
	Td59 (*Triatoma dimidiata*): BAI50847	1	6,724.10	0.81
	Td30 (*Triatoma dimidiata*): BAI50835	2	3,772.83	0.46
	triatin-like salivary lipocalin (*Triatoma infestans*): ABR27935	1	998.66	0.12
	salivary lipocalin (*Triatoma infestans*): ABR27832	1	430.02	0.05
	Td11 (*Triatoma dimidiata*): BAI50818	3	307.67	0.04
	Td124 (*Triatoma dimidiata*): BAI50853	2	35.50	0.00
Clade IV: Triatin-like				
	Pc58 (*Panstrongylus chinai*): BBA30642	3	60,942.16	7.37
	Td18 (*Triatoma dimidiata*): BAI50824	1	4,461.02	0.56
	Pc226 (*Panstrongylus chinai*): BBA30666	3	238.13	0.03
	Td40 (*Triatoma dimidiata*): BAI50840	1	231.81	0.03
	venom triabin-like protein 1 (*Pristhesancus plagipennis*): AQM58444	2	113.57	0.01
	triabin-like protein 2 (*Pristhesancus plagipennis*): AQM58294	2	11.66	0.00
	Pc70 (*Panstrongylus chinai*): BBA30647	1	5.18	0.00
	triabin-like protein 3 (*Pristhesancus plagipennis*): AQM58295	2	5.03	0.00
Clade V: Triafestin-like				
	Td25 (*Triatoma dimidiata*): BAI50830	6	83,453.66	10.09
	Td47 (*Triatoma dimidiata*): BAI50846	5	20,819.94	2.52
	Td27 (*Triatoma dimidiata*): BAI50832	1	10,988.14	1.33
	Pc64 (*Panstrongylus chinai*): BBA30646	1	33.78	0.00
Total		64	827,419.49	100.00

*Data are accessible within the article

## 2 Experimental Design, Materials, and Methods

The sequences of *T. rubrofasciata* salivary lipocalins were obtained in the study “Salivary gland transcriptome of the Asiatic *Triatoma rubrofasciata*” [1]. The trinity sequences coding for the lipocalin family of proteins were aligned with CLUSTAL W software [2] and examined using Molecular Evolutionary Genetics Analysis (MEGA) version 6 [3]. The best maximum likelihood (ML) model for analysis was selected based on the lowest BIC score (Bayesian Information Criterion) in MEGA 6, and phylogenetic trees were constructed by the ML method with the distance algorithms available in the MEGA package. Bootstrap values were determined based on 1,000 replicates of the datasets. Data access is possible by viewing “Salivary gland transcriptome of the Asiatic *Triatoma rubrofasciata*” [1].

## Declaration of Competing Interest

The authors declare that they have no competing financial interests or personal relationships that may have influenced the work reported in this paper.

## Appendix Supplementary materials

Image, application 1

## References

[1] D. Mizushima, A. Tabbabi, D.S. Yamamoto, L.T. Kien, H. Kato, Salivary gland transcriptome of the Asiatic *Triatoma rubrofasciata*. Acta Trop. In press. 10.1016/j.actatropica.2020.105473 32505596

[2] Thompson J.D., Higgins D.G., Gibson T.J. (1994). CLUSTAL W: improving the sensitivity of progressive multiple sequence alignment through sequence weighting, position-specific gap penalties and weight matrix choice. Nucleic Acids Res.

[3] Tamura K., Stecher G., Peterson D., Filipski A., Kumar S. (2013). MEGA6: Molecular Evolutionary Genetics Analysis version 6.0. Mol. Biol. Evol..

