# Supplementary material for: Transcriptome data on salivary lipocalin family of the Asiatic Triatoma rubrofasciata
Source: Data Brief. 2020 Apr 30;30:105647. doi: 10.1016/j.dib.2020.105647 (PMC7214826; doi:10.1016/j.dib.2020.105647)
Supplement: Supplementary file 1 [file mmc1.docx]

Supplementary Table 1.

Accession number and direct link of each molecule

| Accession number | Molecule | direct link |
| --- | --- | --- |
| ICPO01000359 | Tr-Lip58 | http://getentry.ddbj.nig.ac.jp/getentry/na/ICPO01000359/ |
| ICPO01000360 | Tr-Lip55 | http://getentry.ddbj.nig.ac.jp/getentry/na/ICPO01000360/ |
| ICPO01000361 | Tr-Lip13 | http://getentry.ddbj.nig.ac.jp/getentry/na/ICPO01000361/ |
| ICPO01000362 | Tr-Lip60 | http://getentry.ddbj.nig.ac.jp/getentry/na/ICPO01000362/ |
| ICPO01000363 | Tr-Lip45 | http://getentry.ddbj.nig.ac.jp/getentry/na/ICPO01000363/ |
| ICPO01000364 | Tr-Lip40 | http://getentry.ddbj.nig.ac.jp/getentry/na/ICPO01000364/ |
| ICPO01000365 | Tr-Lip32 | http://getentry.ddbj.nig.ac.jp/getentry/na/ICPO01000365/ |
| ICPO01000366 | Tr-Lip57 | http://getentry.ddbj.nig.ac.jp/getentry/na/ICPO01000366/ |
| ICPO01000367 | Tr-Lip18 | http://getentry.ddbj.nig.ac.jp/getentry/na/ICPO01000367/ |
| ICPO01000369 | Tr-Lip22 | http://getentry.ddbj.nig.ac.jp/getentry/na/ICPO01000369/ |
| ICPO01000370 | Tr-Lip54 | http://getentry.ddbj.nig.ac.jp/getentry/na/ICPO01000370/ |
| ICPO01000371 | Tr-Lip35 | http://getentry.ddbj.nig.ac.jp/getentry/na/ICPO01000371/ |
| ICPO01000372 | Tr-Lip31 | http://getentry.ddbj.nig.ac.jp/getentry/na/ICPO01000372/ |
| ICPO01000373 | Tr-Lip09 | http://getentry.ddbj.nig.ac.jp/getentry/na/ICPO01000373/ |
| ICPO01000374 | Tr-Lip19 | http://getentry.ddbj.nig.ac.jp/getentry/na/ICPO01000374/ |
| ICPO01000375 | Tr-Lip52 | http://getentry.ddbj.nig.ac.jp/getentry/na/ICPO01000375/ |
| ICPO01000376 | Tr-Lip14 | http://getentry.ddbj.nig.ac.jp/getentry/na/ICPO01000376/ |
| ICPO01000377 | Tr-Lip36 | http://getentry.ddbj.nig.ac.jp/getentry/na/ICPO01000377/ |
| ICPO01000378 | Tr-Lip43 | http://getentry.ddbj.nig.ac.jp/getentry/na/ICPO01000378/ |
| ICPO01000379 | Tr-Lip51 | http://getentry.ddbj.nig.ac.jp/getentry/na/ICPO01000379/ |
| ICPO01000380 | Tr-Lip46 | http://getentry.ddbj.nig.ac.jp/getentry/na/ICPO01000380/ |
| ICPO01000381 | Tr-Lip56 | http://getentry.ddbj.nig.ac.jp/getentry/na/ICPO01000381/ |
| ICPO01000382 | Tr-Lip08 | http://getentry.ddbj.nig.ac.jp/getentry/na/ICPO01000382/ |
| ICPO01000383 | Tr-Lip25 | http://getentry.ddbj.nig.ac.jp/getentry/na/ICPO01000383/ |
| ICPO01000384 | Tr-Lip24 | http://getentry.ddbj.nig.ac.jp/getentry/na/ICPO01000384/ |
| ICPO01000385 | Tr-Lip04 | http://getentry.ddbj.nig.ac.jp/getentry/na/ICPO01000385/ |
| ICPO01000386 | Tr-Lip16 | http://getentry.ddbj.nig.ac.jp/getentry/na/ICPO01000386/ |
| ICPO01000387 | Tr-Lip11 | http://getentry.ddbj.nig.ac.jp/getentry/na/ICPO01000387/ |
| ICPO01000388 | Tr-Lip29 | http://getentry.ddbj.nig.ac.jp/getentry/na/ICPO01000388/ |
| ICPO01000389 | Tr-Lip34 | http://getentry.ddbj.nig.ac.jp/getentry/na/ICPO01000389/ |
| ICPO01000390 | Tr-Lip27 | http://getentry.ddbj.nig.ac.jp/getentry/na/ICPO01000390/ |
| ICPO01000391 | Tr-Lip23 | http://getentry.ddbj.nig.ac.jp/getentry/na/ICPO01000391/ |
| ICPO01000392 | Tr-Lip10 | http://getentry.ddbj.nig.ac.jp/getentry/na/ICPO01000392/ |
| ICPO01000393 | Tr-Lip44 | http://getentry.ddbj.nig.ac.jp/getentry/na/ICPO01000393/ |
| ICPO01000394 | Tr-Lip50 | http://getentry.ddbj.nig.ac.jp/getentry/na/ICPO01000394/ |
| ICPO01000395 | Tr-Lip02 | http://getentry.ddbj.nig.ac.jp/getentry/na/ICPO01000395/ |
| ICPO01000396 | Tr-Lip41 | http://getentry.ddbj.nig.ac.jp/getentry/na/ICPO01000396/ |
| ICPO01000397 | Tr-Lip07 | http://getentry.ddbj.nig.ac.jp/getentry/na/ICPO01000397/ |
| ICPO01000398 | Tr-Lip59 | http://getentry.ddbj.nig.ac.jp/getentry/na/ICPO01000398/ |
| ICPO01000399 | Tr-Lip62 | http://getentry.ddbj.nig.ac.jp/getentry/na/ICPO01000399/ |
| ICPO01000400 | Tr-Lip06 | http://getentry.ddbj.nig.ac.jp/getentry/na/ICPO01000400/ |
| ICPO01000401 | Tr-Lip15 | http://getentry.ddbj.nig.ac.jp/getentry/na/ICPO01000401/ |
| ICPO01000402 | Tr-Lip05 | http://getentry.ddbj.nig.ac.jp/getentry/na/ICPO01000402/ |
| ICPO01000403 | Tr-Lip49 | http://getentry.ddbj.nig.ac.jp/getentry/na/ICPO01000403/ |
| ICPO01000404 | Tr-Lip63 | http://getentry.ddbj.nig.ac.jp/getentry/na/ICPO01000404/ |
| ICPO01000405 | Tr-Lip53 | http://getentry.ddbj.nig.ac.jp/getentry/na/ICPO01000405/ |
| ICPO01000406 | Tr-Lip42 | http://getentry.ddbj.nig.ac.jp/getentry/na/ICPO01000406/ |
| ICPO01000407 | Tr-Lip21 | http://getentry.ddbj.nig.ac.jp/getentry/na/ICPO01000407/ |
| ICPO01000408 | Tr-Lip37 | http://getentry.ddbj.nig.ac.jp/getentry/na/ICPO01000408/ |
| ICPO01000409 | Tr-Lip30 | http://getentry.ddbj.nig.ac.jp/getentry/na/ICPO01000409/ |
| ICPO01000410 | Tr-Lip38 | http://getentry.ddbj.nig.ac.jp/getentry/na/ICPO01000410/ |
| ICPO01000411 | Tr-Lip17 | http://getentry.ddbj.nig.ac.jp/getentry/na/ICPO01000411/ |
| ICPO01000412 | Tr-Lip26 | http://getentry.ddbj.nig.ac.jp/getentry/na/ICPO01000412/ |
| ICPO01000413 | Tr-Lip64 | http://getentry.ddbj.nig.ac.jp/getentry/na/ICPO01000413/ |
| ICPO01000414 | Tr-Lip39 | http://getentry.ddbj.nig.ac.jp/getentry/na/ICPO01000414/ |
| ICPO01000415 | Tr-Lip28 | http://getentry.ddbj.nig.ac.jp/getentry/na/ICPO01000415/ |
| ICPO01000416 | Tr-Lip20 | http://getentry.ddbj.nig.ac.jp/getentry/na/ICPO01000416/ |
| ICPO01000417 | Tr-Lip47 | http://getentry.ddbj.nig.ac.jp/getentry/na/ICPO01000417/ |
| ICPO01000418 | Tr-Lip48 | http://getentry.ddbj.nig.ac.jp/getentry/na/ICPO01000418/ |
| ICPO01000419 | Tr-Lip61 | http://getentry.ddbj.nig.ac.jp/getentry/na/ICPO01000419/ |
| ICPO01000420 | Tr-Lip12 | http://getentry.ddbj.nig.ac.jp/getentry/na/ICPO01000420/ |
| ICPO01000421 | Tr-Lip03 | http://getentry.ddbj.nig.ac.jp/getentry/na/ICPO01000421/ |
| ICPO01000643 | Tr-Lip33 | http://getentry.ddbj.nig.ac.jp/getentry/na/ICPO01000643/ |
| ICPO01000676 | Tr-Lip01 | http://getentry.ddbj.nig.ac.jp/getentry/na/ICPO01000676/ |
